# Roux-en-Y Gastric Bypass and Caloric Restriction but Not Gut Hormone-Based Treatments Profoundly Impact the Hypothalamic Transcriptome in Obese Rats

**DOI:** 10.3390/nu14010116

**Published:** 2021-12-28

**Authors:** Ulrich Dischinger, Tobias Heckel, Thorsten Bischler, Julia Hasinger, Malina Königsrainer, Angelika Schmitt-Böhrer, Christoph Otto, Martin Fassnacht, Florian Seyfried, Mohammed Khair Hankir

**Affiliations:** 1 Department of Internal Medicine, Division of Endocrinology and Diabetes, University Hospital, University of Wurzburg, 97080 Wurzburg, Germany; Hasinger_J@ukw.de (J.H.); fassnacht_m@ukw.de (M.F.); 2 Core Unit Systems Medicine, University of Wurzburg, 97080 Wurzburg, Germany; heckel.t@web.de (T.H.); thorsten.bischler@uni-wuerzburg.de (T.B.); 3 Department of General, Visceral, Transplant, Vascular and Pediatric Surgery, University Hospital, University of Wurzburg, 97080 Wurzburg, Germany; Koenigsrai_M@ukw.de (M.K.); otto_c@ukw.de (C.O.); seyfried_f@ukw.de (F.S.); Hankir_M@ukw.de (M.K.H.); 4 Department of Psychiatry, Psychosomatics and Psychotherapy, University Hospital, University of Wurzburg, 97080 Wurzburg, Germany; Schmitt_A3@ukw.de

**Keywords:** obesity, Roux-en-Y gastric bypass surgery, liraglutide, PYY3-36, hypothalamic gene expression

## Abstract

Background: The hypothalamus is an important brain region for the regulation of energy balance. Roux-en-Y gastric bypass (RYGB) surgery and gut hormone-based treatments are known to reduce body weight, but their effects on hypothalamic gene expression and signaling pathways are poorly studied. Methods: Diet-induced obese male Wistar rats were randomized into the following groups: RYGB, sham operation, sham + body weight-matched (BWM) to the RYGB group, osmotic minipump delivering PYY3-36 (0.1 mg/kg/day), liraglutide s.c. (0.4 mg/kg/day), PYY3-36 + liraglutide, and saline. All groups (except BWM) were kept on a free choice of high- and low-fat diets. Four weeks after interventions, hypothalami were collected for RNA sequencing. Results: While rats in the RYGB, BWM, and PYY3-36 + liraglutide groups had comparable reductions in body weight, only RYGB and BWM treatment had a major impact on hypothalamic gene expression. In these groups, hypothalamic leptin receptor expression as well as the JAK–STAT, PI3K-Akt, and AMPK signaling pathways were upregulated. No significant changes could be detected in PYY3-36 + liraglutide-, liraglutide-, and PYY-treated groups. Conclusions: Despite causing similar body weight changes compared to RYGB and BWM, PYY3-36 + liraglutide treatment does not impact hypothalamic gene expression. Whether this striking difference is favorable or unfavorable to metabolic health in the long term requires further investigation.

## 1. Introduction

Obesity currently affects more than one-third of the global population and represents a major socioeconomic burden [1,2,3]. Moreover, obesity increases the risk of developing several life-shortening diseases such as type 2 diabetes, chronic heart failure, cardiovascular disease, and cancer [4].

Available noninvasive treatment options for severe obesity lack efficacy and result in long-term weight regain in the vast majority of patients. In contrast, bariatric surgery, in particular, Roux-en-Y gastric bypass (RYGB), achieves marked and sustained weight loss alongside improving metabolic health [5,6,7,8]. The persistently negative energy balance after surgery is considered to be mainly caused by a decrease in energy intake (approximately 50%) [9,10,11,12,13,14,15,16,17,18,19,20,21,22], mostly from fat [9,12,14,17,19,20,22,23,24].

The mechanisms behind the impressive efficacy of RYGB are poorly understood. Previous research concentrated mainly on peripheral effects and, among others, identified markedly increased levels of gut hormones derived from enteroendocrine L-cells suppressing appetite and controlling glucose, such as glucagon-like peptide-1 (GLP-1) and peptide tyrosine tyrosine 3-36 (PYY3-36), in humans and rodent models [25,26,27,28,29,30,31,32,33,34]. We have recently shown that a combinatory treatment ofPYY3-36 and the stable GLP-1 analogue liraglutide leads to similar changes in body weight compared to RYGB in diet-induced obese rats [35]. Both therapies proved to be effective in reducing overall food intake and high-fat preference [35]. Further underlining the relevance of PYY3-36 and GLP-1, we could increase high-fat food preference of RYGB-treated rats by using antagonists of their respective receptors [36].

These findings might suggest GLP-1 + PYY3-36 combination therapy as a pharmaceutical alternative to RYGB for treating obesity. However, perturbing the receptors of PYY3-36 and/or GLP-1 does not weaken the efficacy of RYGB in mice [37,38,39,40,41]. Additionally, plasma levels of many anorexigenic gut hormones such as oxyntomodulin and neurotensin are elevated following RYGB [42,43,44,45,46], which underlines the hormonal complexity behind this surgical intervention.

The hypothalamus is known to be the main integrator of hormonal and neural signals involved in the regulation of food intake and body weight [47,48]. In this brain region, the expression of key orexigenic neuropeptides such as neuropeptide Y (NPY) and agouti-related peptide (AgRP) and anorexigenic neuropeptides such as proopiomelanocortin (POMC) and cocaine- and amphetamine-regulated transcript (CART) is affected by caloric restriction [49,50], RYGB [49,51], and liraglutide [52]. In this study, we wanted to compare the broader effects of these treatments on hypothalamic gene expression by using RNA sequencing.

## 2. Materials and Methods

### 2.1. Animals

Adult male Wistar rats (Charles River Laboratories, *n* = 66, 9–10 weeks old) weighing 328.7 ± 16.3 g, were initially group-housed in a dedicated facility with an ambient room temperature of 22 °C and a 12 h light/dark cycle. They had free access to a high-fat diet (C1090-60 HF diet, 5228 kcal/kg; 60% calories from fat, 16% from protein, and 24% from carbohydrate; Altromin) for 6 weeks to induce obesity, as described before [35]. Animals were then randomized into the following groups with pharmaceutical/surgical treatment: five animals received liraglutide s.c. (0.4 mg/kg/day, Victoza, Novo Nordisk Pharma, Bagsværd, Denmark) and isotonic saline (Braun, Melsungen, Germany) via osmotic minipump. Five animals received PYY3-36 (0.1 mg/kg/day, Hölzel diagnostika, Cologne, Germany) via osmotic minipump and saline s.c. Ten animals received a combination of liraglutide s.c. and PYY3-36 via osmotic minipump, and eight animals received saline only (via osmotic minipump and s.c.). Fourteen animals underwent RYGB, and eleven animals a sham operation. Seven animals were body-weight-matched controls (BWM) by chronic food restriction to induce a similar weight course compared to RYGB animals. This was achieved by restricting the amount of high- and low-fat diet they consumed to that of RYGB animals. Consequently, all groups except BWM had a free choice of high- and low-fat diet (ad libitum) from intervention on (RYGB from the third postoperative day on). Due to capacity reasons, experiments were performed in two different periods (batch A and batch B). However, experiments were performed by the same investigators under the same conditions.

All animal procedures were approved by the local regulatory authority (Regierung von Unterfranken, Würzburg, Germany, AZ: 55.2-2532-2-467). All experiments were performed in accordance with German and European laws and regulations (TierSchG, TierSchVersV, Directive 2010/63/EU). All efforts were made to minimize suffering.

### 2.2. Drugs

We administered liraglutide at a dose of 0.4 mg/kg/day once daily s.c. and PYY3-36 at a dose of 0.1 mg/kg/day [52,53]. PYY3-36 was administered via subcutaneously implanted osmotic minipumps (ALZET pump model 2004) to overcome its short half-life [54]. We administered isotonic saline at a dose of 1 µL/g body weight once daily subcutaneously (corresponding to the volume of liraglutide) and/or via osmotic minipump.

### 2.3. Surgeries

As published before [36], animals were isoflurane-anesthetized and under butorphanol (0.1 mg/kg) analgesia for RYGB and sham operation. A small gastric pouch 5% of the original stomach size was created, and the biliopancreatic and common limbs were made to measure 15 cm and 25 cm in length, respectively [36,55]. After the intervention, animals were transferred to individual cages. Postoperatively, all animals received carprofen (5 mg/kg) for 5 days. From postoperative day 3 onwards, animals (except BWM) were kept on a choice of C1090-10 LF (3514 kcal/kg; 10% calories from fat, 24% from protein, and 66% from carbohydrate; Altromin) and HF diets. LF and HF diet intakes were measured daily for each animal, and HF/LF food preference was calculated as described before.

### 2.4. Extraction of mRNA

Four weeks after intervention, animals were euthanized after 10 h fasting in deep anesthesia. Brains were quickly removed, snap-frozen in liquid nitrogen, and stored at –80 °C until further processing. Using a microscope with an attached freezing plate, hypothalami were isolated from the brains and frozen on dry ice in Eppendorf tubes immediately. The hypothalami were then homogenized using QIAGEN Tissue Lyser II (85300; QIAGEN, Venlo, Netherlands) and further digested using Proteinase K (PK) Solution (MC5005; Promega, Fitchburg, WI, USA). Total RNA was extracted using Promega’s “Maxwell Simply RNA Tissue Kit” (AS1340) and measured using a NanoDrop™ 2000c spectrophotometer (Thermo Fisher Scientific, Waltham, MA, USA).

### 2.5. RNA Sequencing

As described before [56], RNA quality was checked using a 2100 Bioanalyzer with the RNA 6000 Nano kit (Agilent Technologies, Santa Clara, CA, USA). The RIN for all samples was >7.2. DNA libraries suitable for sequencing were prepared from 100 ng of total RNA with oligo-dT capture beads for poly-A-mRNA enrichment using the TruSeq Stranded mRNA Library Preparation Kit (Illumina, San Diego, CA, USA) according to manufacturer’s instructions. After 15 cycles of PCR amplification, the size distribution of the barcoded DNA libraries was estimated ~285/335 bp by electrophoresis on Agilent DNA 1000 Bioanalyzer microfluidic chips.

Sequencing of pooled libraries, spiked with 1% PhiX control library, was performed at 30–50 million reads/sample in single-end mode with 75 nt read length on the NextSeq 500 platform (Illumina) with High Output Kits v2.5. Demultiplexed FASTQ files were generated with bcl2fastq2 v2.20.0.422 (Illumina).

To assure high sequence quality, Illumina reads were quality- and adapter-trimmed via Cutadapt version 1.16/2.1/2.5 using a cutoff Phred score of 20 in NextSeq mode, and reads without any remaining bases were discarded (command line parameters: --nextseq-trim = 20 -m 1 -a AGATCGGAAGAGCACACGTCTGAACTCCAGTCAC). Processed reads were subsequently mapped to the rat genome (GCF_000001895.5/Rnor_6.0 primary assembly and mitochondrion) using STAR v2.7.2b with default parameters based on RefSeq annotation version 106 for GCF_000001895.5/Rnor_6.0 [57]. Read counts on exon level summarized for each gene were generated using featureCounts v1.6.4 from the Subread package [58]. Multi-mapping and multi-overlapping reads were counted strand-specific and reversely stranded with a fractional count for each alignment and overlapping feature (command line parameters: -s 2 -t exon -M -O --fraction). The count output was utilized to identify differentially expressed genes using DESeq2 [59] version 1.24.0. Read counts were normalized by DESeq2, and fold-change shrinkage was applied by setting the parameter “betaPrior = TRUE”. Differential expression of genes was assumed at an adjusted *p*-value (padj) after Benjamini–Hochberg correction <0.05 and |log2FoldChange| > 1. Considering the DESeq2 log2 fold change of all analyzed genes, clusterProfiler [60] version 3.12.0 was used to perform gene set enrichment analysis (GSEA) based on Kyoto Encyclopedia of Genes and Genomes (KEGG) pathways.

### 2.6. Quantitative Real-Time PCR

Total RNA samples (see Section 2.4) were reverse-transcribed into cDNA using QuantiTect Reverse Transcription Kit (205311; QIAGEN) and Eppendorf Mastercycler Gradient Instrument. CDNA was stored at −20 °C before further use. Quantitative real-time PCR (qPCR) was performed (samples in duplicates) to verify RNA sequencing results using gene-specific primer and hydrolysis probes (*Lepr*: Rn01433205_m1, NM_012596.1; TaqMan^®^; Thermo Fisher Scientific) on a CFX96™ Real-Time PCR Detection System. Efficiency and Cq-values of samples were calculated using LinRegPCR software (v2020.0). Efficiency-correction and normalization to reference genes (*Actb* (Rn00667869_m1, NM_031144.3), *Ubc* (Rn01499642_m1, BC103477.1)) were performed using qBase+ (Biogazelle).

### 2.7. Blood Sampling and Enzyme-Linked Immunosorbent Assay

Plasma samples were collected under fasting conditions and twenty minutes following oral consumption of a mixed meal under deep anesthesia shortly prior euthanasia. Immediately after collection in tubes pretreated with a DPP-IV inhibitor (Merck), plasma was separated from the blood samples by centrifugation at 5 × 103 rpm for 10 min at 4 °C and stored at −80 °C. To measure GLP-1 and PYY levels (all samples in duplicates), rat-specific enzyme immunoassay/enzyme-linked immunosorbent (EIA/ELISA) kits were used (EK-028-11 and EK-059-04(Phoenix Pharmaceuticals)).

### 2.8. Statistical Analysis

Two-tailed unpaired t-test and two-way ANOVA (repeated measures) with Tukey’s post hoc comparison test where appropriate were used for statistical analysis using GraphPad Prism (version 8.1.2) software. Due to mRNA extraction error, one PYY-treated animal had to be excluded from the analyses.

## 3. Results

### 3.1. RYGB and PYY3-36 + Liraglutide Lead to Similar Changes in Body Weight

**Figure 1:**
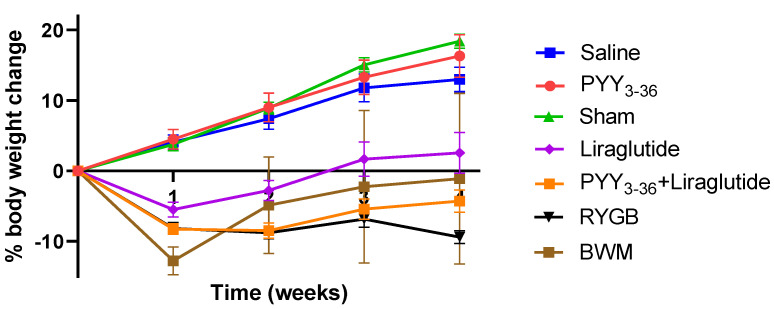
RYGB and PYY3-36 + liraglutide induce body weight loss in diet-induced obese Wistar rats. Body weight in percent (%) weight change from baseline (intervention) of RYGB (*n* = 14), sham (*n* = 11), PYY3-36 + liraglutide (*n* = 10), liraglutide (*n* = 5), PYY3-36 (*n* = 5), BWM (*n* = 7), and saline (*n* = 8) groups. Significant differences between RYGB and sham as well as between PYY3-36 + liraglutide and saline from week one on (*p* ≤ 0.0001). Data are presented as mean ± standard error of the mean.

On the day of surgery and start of medical treatment, respectively, animals of the different treatment/control groups had similar body weights (476.1 ± 8.8 g for the RYGB group, 482.0 ± 12.6 g for the sham group, 545.3 ± 21.3 g for the PYY3-36 + liraglutide group, 500.5 ± 9.5 g for the PYY3-36 group, 487.6 ± 9.6 g for the liraglutide group, 530.4 ± 20.7 g for the saline group, 522.8 ± 16.2 for the low-fat-diet-only group, and 488.0 ± 14.5 g for the BWM group). Post intervention, sham-, saline-, and PYY3-36-treated animals gained weight continuously, while RYGB-, PYY3-36 + liraglutide-, and liraglutide-treated animals began to lose weight. RYGB- and PYY3-36 + liraglutide-treated animals presented with a achieved sustained and plateaued weight loss (effect of intervention: F(6, 58) = 46.5, *p* ≤ 0.0001; effect of time: F(4, 232) = 66.1, *p* ≤ 0.0001; interaction: F(24, 232) = 22.03, *p* ≤ 0.0001, Figure 1). Post hoc testing revealed significant differences between RYGB and sham as well as between PYY3-36 + liraglutide and saline from week one on (*p* ≤ 0.0001). There was a significant difference between RYGB and BWM only in the last week of the observation period (*p* ≤ 0.001).

### 3.2. RYGB and PYY3-36 + Liraglutide Lower Overall Food Intake and Preference for High Fat Diet

As published before (35), RYGB, PYY3-36 + liraglutide, and liraglutide reduced overall food intake (in kcal) in the observation period (effect of intervention: F(5, 300) = 75.2, *p* ≤ 0.0001; effect of time: F(5, 300) = 17.3, *p* ≤ 0.0001; interaction: F(25, 300) = 3.3, *p* ≤ 0.0001).

### 3.3. RYGB and PYY3-36 + Liraglutide Increase Plasma Levels of GLP-1

Fasted GLP-1 levels were significantly higher in liraglutide-treated animals compared to saline-treated animals (F(3, 13) = 6.3, *p* ≤ 0.01. Post hoc: *p* ≤ 0.05). As shown before [60], meal-induced GLP-1 release compared to fasting GLP-1 release was significantly higher in RYGB-treated animals than in sham-treated animals (0.47 ± 0.18 ng/mL vs. 0.80 ± 0.22 ng/mL. F(7, 26) = 4.4, *p* ≤ 0.01. Post hoc: *p* ≤ 0.05). Regarding levels of PYY 3-36, there was also a clear tendency (RYGB: 0.55 ± 0.37 ng/mL vs. sham: 0.27 ± 0.06 ng/mL. F(7, 25) = 2.8, *p* ≤ 0.05. Post hoc: *p* = 0.25).

### 3.4. Only RYGB and Food Restriction Impact Hypothalamic mRNA Expression

**Figure 2:**
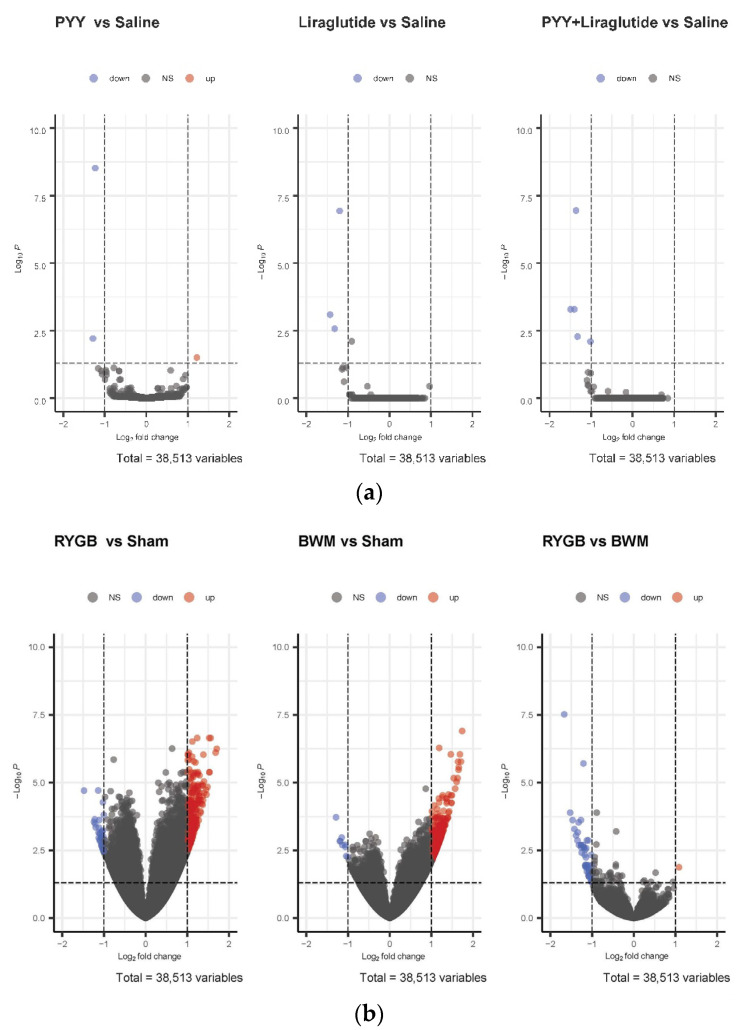
(**a**) Volcano plots of differentially expressed genes in the hypothalami of PYY- vs. saline-treated animals, liraglutide- vs. saline-treated animals and PYY3-36 + liraglutide- vs. saline-treated animals. (**b**) Volcano plots of differentially expressed genes in hypothalami of RYGB- vs. sham-treated animals, BWM vs. sham-treated animals and RYGB-treated vs. BWM treated animals.

While no relevant differences in hypothalamic mRNA expression were found in PYY3-36-, PYY3-36 + liraglutide-, and liraglutide-treated animals compared with saline controls (Figure 2a), a high number of genes were regulated in RYGB-treated animals (266 genes upregulated) and BWM animals (326 genes upregulated) compared with sham controls (Figure 2b).

**Figure 3:**
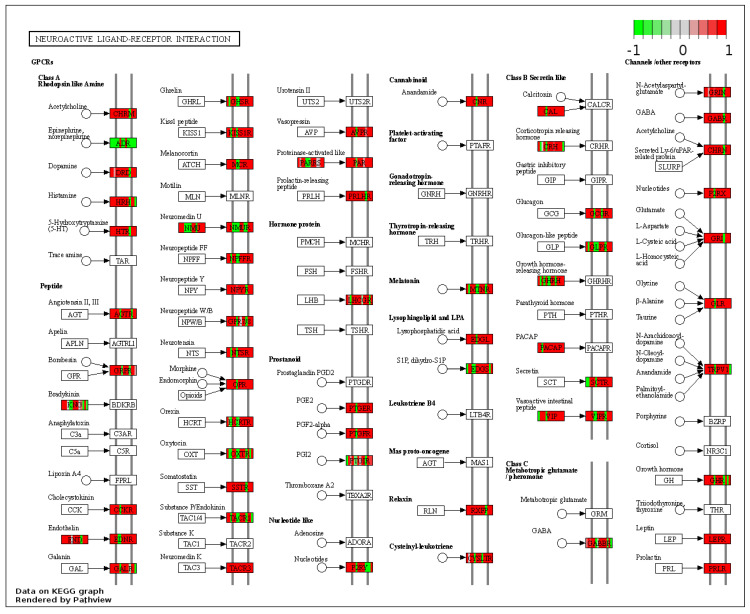
Kyoto Encyclopedia of Genes and Genomes (KEGG) pathway “Neuroactive ligand–receptor interaction” (RYGB vs. sham). Higher expressed genes in red, and lower expressed genes in green. Data rendered by Pathview (https://pathview.uncc.edu/, accessed on 27 December 2021).

These genes could be attached to a significant number of pathways identified as up-regulated by gene-set enrichment analysis (see Tables S1 and S2). In RYGB- vs. sham-treated animals, the pathway for neuroactive ligand–receptor interaction was upregulated (Figure 3) and oxidative phospohorylation downregulated.

**Figure 4:**
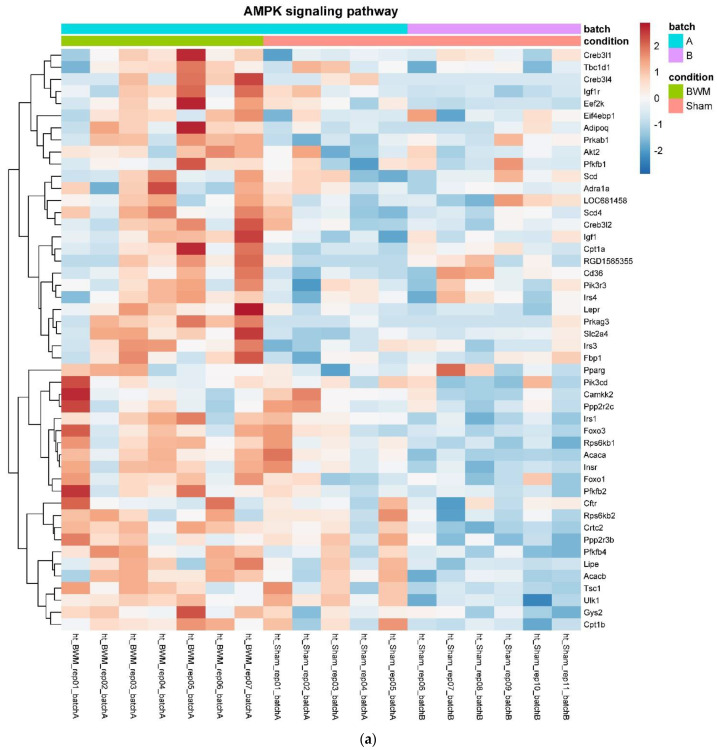

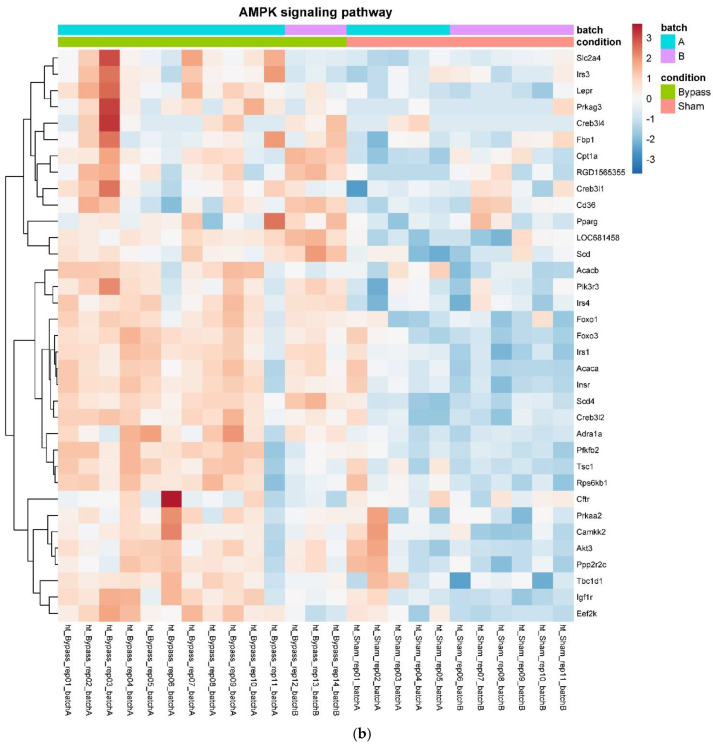

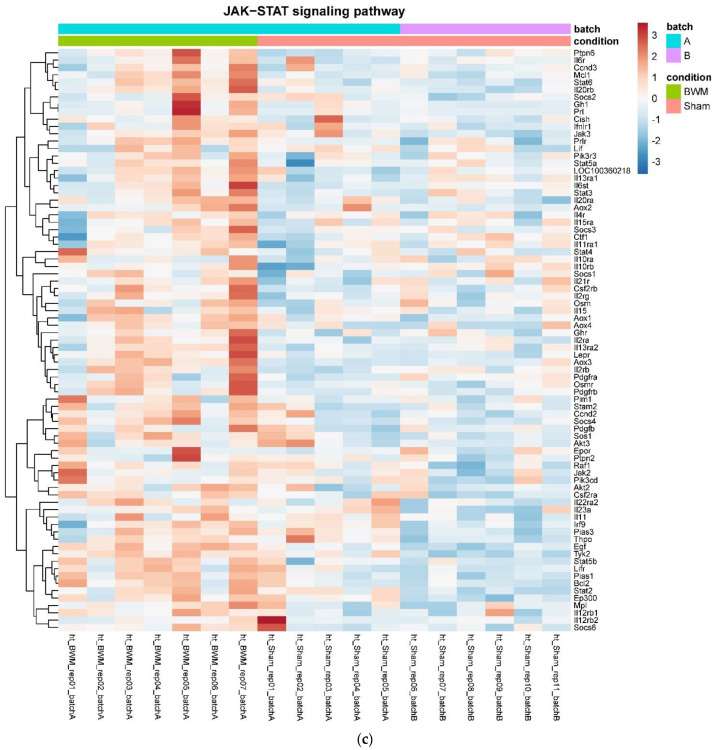

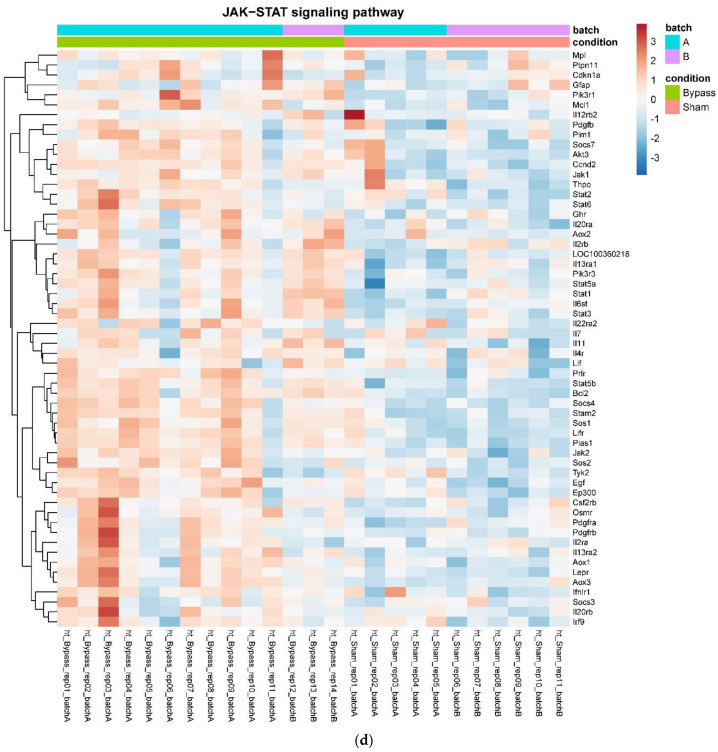

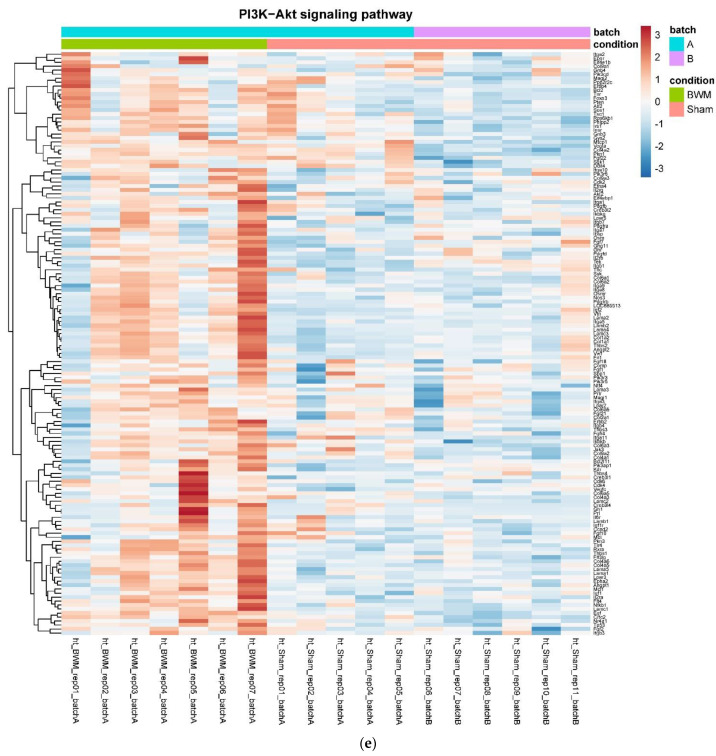

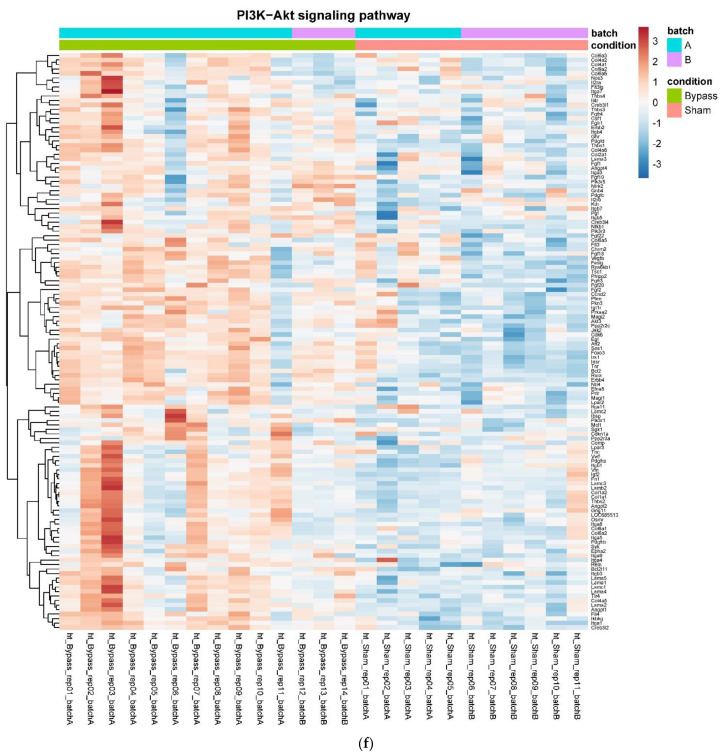
Heatmaps showing gene expression differences between treatment and control samples for the GSEA core enrichment of the pathways AMPK (**a**,**b**), JAK–STAT (**c**,**d**), PI3K–Akt (**e**,**f**) in BWM vs. sham and RYGB- vs. sham-treated animals. Analysis was performed based on DESeq2 log2 fold changes of all analyzed genes (without applying any filters). Expression values represent row-wise z-scores of vst-transformed read counts with higher expressed genes in red and lower expressed genes in blue. GESA, gene set enrichment analysis.

In detail, when analyzing normalized counts of genes of these pathways, we found significantly higher levels of *Htr1b*, *Htr2a*, *Htr5a*, *Glra1*, *Glra3*, *Glra4*, *Npy4r*, *Mc3r*, *Mc4r*, *Prlr*, and *Insr* and significantly lower levels of *Sdhb, Sdhc*, and *Sdhd* in RYGB- vs. Sham-treated animals. Looking for similar differences in RYGB and BWM groups compared with the sham group, we identified an upregulation of the leptin receptor (*Lepr*) and its mediating pathways, such as the JAK–STAT, the PI3K–Akt, and the AMPK pathway (Figure 4). Additionally, we directly compared RYGB-treated and BWM treated animals. This revealed 48 genes, which were downregulated in RYGB-treated vs. BWM treated animals (one upregulated gene). We identified four genes connected to the regulation of food intake: *Ffar4, Ucn, Tph2,* and *Mrap.* Comparing normalized counts of these genes revealed no significant differences. Accordingly, pathway analysis generated no conclusive results.

Furthermore, we found higher counts of *Lepr* in RYGB-treated and BWM treated animals than in sham (F(2, 29) = 3.5, *p* ≤ 0.05), which could be confirmed by qPCR. Here, *Lepr* was significantly higher expressed in RYGB- than in sham-treated animals (0.5 ± 0.2 vs. 0.25 ± 0.13, *p* ≤ 0.05). Notably, there were also relevant differences in hypothalamic mRNA expression in RYGB-treated compared with BWM animals (Figure 2b).

## 4. Discussion

RYGB is one of the most effective treatment options for obesity, but its central mechanisms of action are poorly understood. It is also unknown how anorexigenic gut hormones and their analogues such as liraglutide, which is used in clinical practice to treat obesity, impact the hypothalamic transcriptome.

Using a hypothesis-free RNA sequencing method, we found that only RYGB and BWM rats induced relevant differences in hypothalamic mRNA expression compared with controls. Although equally effective as RYGB in terms of body weight loss and suppression of food intake, treatment with PYY3-36 + liraglutide led to no relevant changes in hypothalamic mRNA expression compared with controls. The same was the case for PYY3-36 and liraglutide treatments individually. Therefore, no upregulated pathways (as in earlier studies with liraglutide, e.g., PI3K/AKT or MAPK [61]) could be identified. While these results are somewhat unexpected, they are in line with those from a recent metabolomics study in obese and diabetic/prediabetic patients showing that RYGB and 4 weeks caloric restriction, but not a gut hormone combination therapy (GLP-1 + oxyntomodulin + PYY), exerted profound effects in urinary and plasma metabolites [62]. We unfortunately could not perform such a metabolomics analysis in the present study due to lack of available samples. However, it is possible that the changes in plasma metabolites present after RYGB and BWM, but not after PYY3-36 + liraglutide, are responsible for the detected changes in hypothalamic gene expression. This is supported by the profound changes we previously found in plasma metabolites in RYGB and BWM compared with Sham Zucker Fatty rats [63].

When comparing results in RYGB and BWM animals with sham animals, there was a clear overlap in leptin receptor signaling. We found that leptin receptor expression (normalized counts and mRNA) as well as JAK–STAT, PI3K–Akt and AMPK signaling pathways [47,64] were consistently upregulated in RYGB-treated and BWM treated rats, suggesting that both treatments enhance hypothalamic leptin sensitivity. This contrasts with the findings of a recent study in which leptin signaling and effects on food intake were enhanced in RYGB but not in BWM rats due to a reduction in hypothalamic gliosis and inflammation [65]. Our findings are in line, however, with those of Barkholt et al. [49] showing that both RYGB and BWM rats have similar effects on hypothalamic gene expression and that both interventions reduce markers of hypothalamic gliosis and inflammation.

Unlike weight loss from caloric restriction, RYGB does result in powerful counter-regulatory responses such as increased hunger that promote weight regain. It would therefore be expected that RYGB induces unique effects on hypothalamic gene expression not seen in BWM rats. Indeed, there were significant changes in neuroactive ligand–receptor interaction in RYGB rats. These included an upregulation of receptors with established roles in promoting a negative energy balance, such as the CCKR, NTSR, VIPR, MCR, and CHRN. Also only detectable in hypothalami of RYGB-treated animals was a downregulation of genes related to the oxidative phosphorylation pathway. This reflects most probably the improved hypothalamic mitochondrial function in these animals The direct comparison of the groups RYGB and BWM provided a relevant number of differentially regulated genes. Most of these genes were downregulated in RYGB-treated compared to BWM treated animals. Analyzing genes involved in the regulation of food intake (*Ffar4, Ucn, Tph2*, and *Mrap*) revealed, however, no significant differences. Accordingly, pathway analysis was not conclusive. Future studies have to go more into detail comparing hypothalamic effects of RYGB vs. diet-induced weight loss.

This work has several limitations. First of all, we only performed RNA analyses and no functional analyses. Second, due to methodological reasons, we were not able to differentiate between the different hypothalamic nuclei. Third, while surgical groups were larger in number due to a higher expected but fortunately not met drop-out rate, the groups of animals treated with liraglutide or PYY3-36 consisted of only five animals each. In-group consistency regarding body weight and food intake was strong, however, and in previous RYGB studies we clearly experienced that a number of five animals per group is generally sufficient to reach adequate power [66,67,68]. Due to the overall high number of animals, it was not possible to start the individual treatment of all animals on the same day after randomization. Because of this, RYGB-treated animals had initially significantly lower body weights than those of the animals of the PYY3-36 + liraglutide group. This surely weakens the comparability of the groups to some extent; however, it has to be stated that a similar body weight change in percent should still show the comparable effectivity of the treatments. As an additional limitation, BWM treated animals were heavier than RYGB-treated animals in the end of the observation period.

In summary, we demonstrated in a direct head-to-head comparison, that a combination of PYY3-36 and liraglutide treatment can have similar effects on body weight and food intake compared to RYGB and caloric restriction, but that only the latter treatments impact hypothalamic gene expression. In other words, while the outward effects of gut hormone-based treatments on metabolic disease appear similar to those of surgical and dietary interventions, the central effects are vastly different. Whether this in turn is favorable or unfavorable to metabolic health in the long term requires further investigation.

## Data Availability

RNA-Sequencing data is accessible here: https://www.ncbi.nlm.nih.gov/geo/query/acc.cgi?acc=GSE190218 (last accessed on 27 December 2021). A token (for reviewers) can be obtained from the corresponding author.

## Supplementary Materials

The following are available online at https://www.mdpi.com/article/10.3390/nu14010116/s1, https://github.com/Dischinger/https-github.com-Dischinger-Roux-en-Y-gastric-bypass-and-caloric-restriction.git: Table S1: Results of KEGG pathway mapping on the basis of the comparison of hypothalamic mRNA results in RYGB- vs. sham-treated animals. Pathways with adjusted *p*-value ≤ 0.05 only. Table S2: Results of KEGG pathway mapping on the basis of the comparison of hypothalamic mRNA results in BWM vs. sham-treated animals. Pathways with adjusted *p*-values ≤ 0.05 only.
