# Supplementary material for: Roux-en-Y Gastric Bypass and Caloric Restriction but Not Gut Hormone-Based Treatments Profoundly Impact the Hypothalamic Transcriptome in Obese Rats
Source: Nutrients. 2021 Dec 28;14(1):116. doi: 10.3390/nu14010116 (PMC8746874; doi:10.3390/nu14010116)
Supplement: Supplementary file 1 [file nutrients-14-00116-s001.zip › nutrients-1520618-supplementary.pdf]

Table S1. Results of KEGG pathway mapping on the basis of the comparison of hypothalamic mRNA results in RYGB vs. sham treated animals. Pathways with p.adjusted≤ 0.05 only.

ID	Description	setSize	enrichmentScore	NES	pvalue	p.adjust	qvalues	rank
rno05168	Herpes simplex virus 1 infection	376	0,425142	1,533654	0,001037	0,01752	0,011973	8704
rno05165	Human papillomavirus infection	345	0,469482	1,681053	0,001049	0,01752	0,011973	7039
rno04151	PI3K-Akt signaling pathway	341	0,459294	1,654099	0,001054	0,01752	0,011973	6810
rno04080	Neuroactive ligand-receptor interaction	339	0,456102	1,647899	0,001072	0,01752	0,011973	4716
rno05206	MicroRNAs in cancer	251	0,420367	1,483651	0,001089	0,01752	0,011973	8712
rno05205	Proteoglycans in cancer	204	0,47872	1,646001	0,00111	0,01752	0,011973	7056
rno04022	cGMP-PKG signaling pathway	169	0,429261	1,467395	0,001114	0,01752	0,011973	6337
rno04621	NOD-like receptor signaling pathway	172	0,481805	1,667434	0,001115	0,01752	0,011973	7825
rno04510	Focal adhesion	200	0,547252	1,89303	0,00112	0,01752	0,011973	6172
rno04514	Cell adhesion molecules	163	0,470979	1,587315	0,001135	0,01752	0,011973	8774
rno04390	Hippo signaling pathway	157	0,443773	1,50621	0,001147	0,01752	0,011973	6643
rno04934	Cushing syndrome	155	0,442055	1,494414	0,001151	0,01752	0,011973	7978
rno05224	Breast cancer	146	0,467044	1,574252	0,001164	0,01752	0,011973	6602
rno04630	JAK-STAT signaling pathway	152	0,505769	1,713026	0,001174	0,01752	0,011973	7855
rno04668	TNF signaling pathway	110	0,512368	1,686079	0,001189	0,01752	0,011973	8703
rno04974	Protein digestion and absorption	102	0,596591	1,930475	0,001215	0,01752	0,011973	4942
rno04933	AGE-RAGE signaling pathway in diabetic complications	101	0,519177	1,697643	0,001238	0,01752	0,011973	7686
rno05146	Amoebiasis	95	0,512213	1,655097	0,001245	0,01752	0,011973	6113

rno04610	Complement and coagulation cascades	84	0,641777	1,999966	0,001248	0,01752	0,011973	3706
rno05145	Toxoplasmosis	111	0,513113	1,680863	0,001253	0,01752	0,011973	8325
rno04512	ECM-receptor interaction	87	0,652148	2,081489	0,001282	0,01752	0,011973	6576
rno04927	Cortisol synthesis and secretion	66	0,544918	1,662977	0,0013	0,01752	0,011973	8486
rno04920	Adipocytokine signaling pathway	72	0,574229	1,792615	0,001316	0,01752	0,011973	5760
rno05144	Malaria	55	0,549532	1,629942	0,001344	0,01752	0,011973	4623
rno04340	Hedgehog signaling pathway	52	0,628368	1,848361	0,001348	0,01752	0,011973	6623
rno04392	Hippo signaling pathway - multiple species	28	0,672378	1,76493	0,001439	0,017986	0,012291	6643
rno04020	Calcium signaling pathway	237	0,421177	1,488492	0,002165	0,02415	0,016504	5942
rno04060	Cytokine-cytokine receptor interaction	257	0,431851	1,51911	0,002165	0,02415	0,016504	7877
rno05164	Influenza A	168	0,434239	1,478893	0,002237	0,02415	0,016504	8869
rno04935	Growth hormone synthesis, secretion and action	116	0,487073	1,599614	0,002392	0,02415	0,016504	7039
rno04928	Parathyroid hormone synthesis, secretion and action	105	0,465182	1,503843	0,00241	0,02415	0,016504	5910
rno04931	Insulin resistance	109	0,495663	1,643606	0,002418	0,02415	0,016504	8703
rno04064	NF-kappa B signaling pathway	98	0,50506	1,635355	0,002494	0,02415	0,016504	8868
rno04620	Toll-like receptor signaling pathway	93	0,505008	1,629888	0,002558	0,02415	0,016504	8868
rno04917	Prolactin signaling pathway	72	0,521043	1,623198	0,002601	0,02415	0,016504	7231
rno05321	Inflammatory bowel disease	59	0,543141	1,615396	0,002774	0,025042	0,017114	4750

rno04550	Signaling pathways regulating pluripotency of stem cells	141	0,437983	1,478774	0,003517	0,030255	0,020676	8022
rno04152	AMPK signaling pathway	123	0,475658	1,580248	0,003538	0,030255	0,020676	5603
rno04211	Longevity regulating pathway	89	0,476845	1,520762	0,003699	0,030255	0,020676	8703
rno00830	Retinol metabolism	79	0,495718	1,550117	0,003764	0,030255	0,020676	3965
rno03050	Proteasome	47	-0,65356	-2,16626	0,003817	0,030255	0,020676	9511
rno02010	ABC transporters	50	0,570979	1,675837	0,004082	0,030905	0,021121	5700
rno04930	Type II diabetes mellitus	47	0,542947	1,595419	0,004144	0,030905	0,021121	6445
rno00970	Aminoacyl-tRNA biosynthesis	67	-0,59165	-2,1272	0,004184	0,030905	0,021121	3788
rno04010	MAPK signaling pathway	291	0,375062	1,330208	0,004315	0,031164	0,021297	7510
rno04910	Insulin signaling pathway	138	0,429325	1,431453	0,004662	0,032938	0,02251	7510
rno04926	Relaxin signaling pathway	127	0,445325	1,474672	0,005945	0,039634	0,027086	7997
rno03040	Spliceosome	134	-0,50703	-2,04114	0,006024	0,039634	0,027086	8414
rno01200	Carbon metabolism	122	-0,3628	-1,4302	0,006098	0,039634	0,027086	9623
rno04350	TGF-beta signaling pathway	94	0,477579	1,537428	0,006098	0,039634	0,027086	8173
rno00190	Oxidative phosphorylation	125	-0,71278	-2,83863	0,006711	0,042769	0,029228	5314
rno04932	Non-alcoholic fatty liver disease	151	-0,45637	-1,86695	0,006993	0,043706	0,029869	4624
rno04723	Retrograde endocannabinoid signaling	144	-0,41259	-1,66433	0,007813	0,047907	0,03274	5547
rno04310	Wnt signaling pathway	161	0,415621	1,40927	0,008102	0,048761	0,033323	7978

Table S2. Results of KEGG pathway mapping on the basis of the comparison of hypothalamic mRNA results in BWM vs. sham treated animals. Pathways with p.adjusted ≤ 0.05 only.

ID	Description	setSize	enrichmentScore	NES	pvalue	p.adjust	qvalues	rank

rno04151	PI3K-Akt signaling pathway	341	0,537428	1,764344	0,001004	0,007767	0,004603	6684
rno05168	Herpes simplex virus 1 infection	376	0,500028	1,634425	0,001004	0,007767	0,004603	8921
rno04010	MAPK signaling pathway	291	0,460442	1,487375	0,001007	0,007767	0,004603	6651
rno05165	Human papillomavirus infection	345	0,535059	1,760798	0,001007	0,007767	0,004603	6755
rno05166	Human T-cell leukemia virus 1 infection	238	0,489225	1,576963	0,00101	0,007767	0,004603	6679
rno04060	Cytokine-cytokine receptor interaction	257	0,5784	1,843946	0,001013	0,007767	0,004603	4717
rno05205	Proteoglycans in cancer	204	0,535253	1,720996	0,001013	0,007767	0,004603	6797
rno05206	MicroRNAs in cancer	251	0,464661	1,491083	0,001017	0,007767	0,004603	6904
rno04510	Focal adhesion	200	0,601001	1,928159	0,001022	0,007767	0,004603	6333
rno04621	NOD-like receptor signaling pathway	172	0,522381	1,671006	0,001022	0,007767	0,004603	8921
rno05169	Epstein-Barr virus infection	219	0,501604	1,62225	0,001026	0,007767	0,004603	6616
rno05202	Transcriptional misregulation in cancer	186	0,506337	1,629042	0,001032	0,007767	0,004603	7905
rno05152	Tuberculosis	176	0,48629	1,533782	0,001036	0,007767	0,004603	5091
rno04390	Hippo signaling pathway	157	0,487535	1,526583	0,001041	0,007767	0,004603	3570
rno04934	Cushing syndrome	155	0,505241	1,592152	0,001042	0,007767	0,004603	6317
rno05164	Influenza A	168	0,531133	1,672794	0,001045	0,007767	0,004603	8975
rno04630	JAK-STAT signaling pathway	152	0,617805	1,946985	0,001048	0,007767	0,004603	8765
rno04550	Signaling pathways regulating pluripotency of stem cells	141	0,484002	1,519709	0,00105	0,007767	0,004603	8235
rno04514	Cell adhesion molecules	163	0,557336	1,75251	0,001052	0,007767	0,004603	4748
rno05162	Measles	142	0,495308	1,55334	0,001054	0,007767	0,004603	6890
rno04668	TNF signaling pathway	110	0,603409	1,858053	0,001059	0,007767	0,004603	7059
rno05224	Breast cancer	146	0,521456	1,642849	0,00106	0,007767	0,004603	6852

rno04380	Osteoclast differentiation	121	0,513122	1,590528	0,001063	0,007767	0,004603	7905
rno04659	Th17 cell differentiation	102	0,584622	1,783785	0,001064	0,007767	0,004603	4634
rno04611	Platelet activation	126	0,520243	1,616135	0,001073	0,007767	0,004603	3804
rno05145	Toxoplasmosis	111	0,619462	1,896353	0,001079	0,007767	0,004603	5571
rno04064	NF-kappa B signaling pathway	98	0,625851	1,907894	0,001082	0,007767	0,004603	5941
rno04928	Parathyroid hormone synthesis, secretion and action	105	0,513293	1,571499	0,001085	0,007767	0,004603	5017
rno04933	AGE-RAGE signaling pathway in diabetic complications	101	0,59577	1,821338	0,001085	0,007767	0,004603	7284
rno05410	Hypertrophic cardiomyopathy	91	0,554283	1,676396	0,001088	0,007767	0,004603	6664
rno04974	Protein digestion and absorption	102	0,713025	2,169708	0,001091	0,007767	0,004603	5259
rno04658	Th1 and Th2 cell differentiation	88	0,60828	1,82666	0,001103	0,007767	0,004603	4634
rno05146	Amoebiasis	95	0,640747	1,929177	0,001103	0,007767	0,004603	4745
rno04612	Antigen processing and presentation	80	0,59192	1,761336	0,001106	0,007767	0,004603	6446
rno05414	Dilated cardiomyopathy	94	0,540239	1,631458	0,001107	0,007767	0,004603	6664
rno04512	ECM-receptor interaction	87	0,729915	2,194053	0,001109	0,007767	0,004603	5391
rno04657	IL-17 signaling pathway	87	0,563089	1,682937	0,001111	0,007767	0,004603	5427
rno05222	Small cell lung cancer	92	0,5863	1,796336	0,001111	0,007767	0,004603	6235
rno04640	Hematopoietic cell lineage	89	0,564301	1,681782	0,001112	0,007767	0,004603	7230
rno04061	Viral protein interaction with cytokine and cytokine receptor	83	0,594567	1,758444	0,001114	0,007767	0,004603	4482
rno05150	Staphylococcus aureus infection	87	0,581628	1,759024	0,001115	0,007767	0,004603	7228
rno04620	Toll-like receptor signaling pathway	93	0,62841	1,89632	0,001116	0,007767	0,004603	6565
rno05416	Viral myocarditis	78	0,548204	1,620212	0,001122	0,007767	0,004603	4748

rno04920	Adipocytokine signaling pathway	72	0,565743	1,6636	0,00113	0,007767	0,004603	5051
rno04610	Complement and coagulation cascades	84	0,669845	1,987684	0,001131	0,007767	0,004603	6387
rno05320	Autoimmune thyroid disease	64	0,607289	1,764908	0,001152	0,007767	0,004603	6446
rno05144	Malaria	55	0,615522	1,727209	0,001157	0,007767	0,004603	5051
rno05321	Inflammatory bowel disease	59	0,70089	2,004387	0,001159	0,007767	0,004603	4634
rno05330	Allograft rejection	55	0,594561	1,68215	0,001171	0,007767	0,004603	6446
rno02010	ABC transporters	50	0,596395	1,66345	0,001206	0,007841	0,004647	8489
rno04392	Hippo signaling pathway - multiple species	28	0,699705	1,785772	0,001292	0,008233	0,00488	5572
rno05163	Human cytomegalovirus infection	241	0,448945	1,462735	0,002022	0,012508	0,007413	6852
rno04145	Phagosome	177	0,465163	1,46771	0,002068	0,012508	0,007413	6056
rno05161	Hepatitis B	157	0,468252	1,479785	0,002088	0,012508	0,007413	6855
rno04935	Growth hormone synthesis, secretion and action	116	0,507444	1,556563	0,002144	0,012508	0,007413	7284
rno04670	Leukocyte transendothelial migration	115	0,488233	1,509959	0,002155	0,012508	0,007413	6358
rno00830	Retinol metabolism	79	0,533592	1,567589	0,002237	0,012597	0,007466	5003
rno05140	Leishmaniasis	71	0,566777	1,645512	0,002288	0,012597	0,007466	6258
rno04213	Longevity regulating pathway - multiple species	63	0,544908	1,572501	0,002296	0,012597	0,007466	6755
rno05332	Graft-versus-host disease	54	0,585034	1,667987	0,002326	0,012597	0,007466	6446
rno04020	Calcium signaling pathway	237	0,425547	1,360698	0,003036	0,015401	0,009128	6674
rno04015	Rap1 signaling pathway	210	0,441293	1,416363	0,003049	0,015401	0,009128	7840
rno04926	Relaxin signaling pathway	127	0,490415	1,534576	0,003158	0,015401	0,009128	4634
rno04270	Vascular smooth muscle contraction	138	0,463445	1,455451	0,003168	0,015401	0,009128	3869
rno05135	Yersinia infection	135	0,467803	1,467379	0,003168	0,015401	0,009128	6565

rno04931	Insulin resistance	109	0,503924	1,542625	0,003191	0,015401	0,009128	7905
rno05322	Systemic lupus erythematosus	115	0,491905	1,511976	0,003209	0,015401	0,009128	6869
rno04350	TGF-beta signaling pathway	94	0,525836	1,588862	0,003222	0,015401	0,009128	7911
rno05323	Rheumatoid arthritis	83	0,53314	1,568723	0,003344	0,015753	0,009337	4669
rno05217	Basal cell carcinoma	61	0,552056	1,598062	0,003488	0,016064	0,009521	8235
rno04672	Intestinal immune network for IgA production	43	0,622163	1,699773	0,003521	0,016064	0,009521	6638
rno04622	RIG-I-like receptor signaling pathway	61	0,555518	1,591503	0,003559	0,016064	0,009521	9385
rno05167	Kaposi sarcoma-associated herpesvirus infection	210	0,437495	1,410547	0,004082	0,017943	0,010635	6979
rno04022	cGMP-PKG signaling pathway	169	0,455042	1,43711	0,004128	0,017943	0,010635	7048
rno04310	Wnt signaling pathway	161	0,449751	1,413204	0,004141	0,017943	0,010635	6224
rno00020	Citrate cycle (TCA cycle)	31	-0,64583	-2,02634	0,004329	0,018331	0,010865	8639
rno05142	Chagas disease	103	0,501151	1,519947	0,004343	0,018331	0,010865	6549
rno01522	Endocrine resistance	94	0,492994	1,498865	0,004469	0,018511	0,010972	7284
rno04917	Prolactin signaling pathway	72	0,538376	1,587757	0,004525	0,018511	0,010972	7905
rno05133	Pertussis	73	0,542703	1,591595	0,004561	0,018511	0,010972	6834
rno04927	Cortisol synthesis and secretion	66	0,569957	1,658347	0,004614	0,018511	0,010972	6317
rno04614	Renin-angiotensin system	32	0,620398	1,615778	0,004957	0,019645	0,011644	5085
rno05310	Asthma	25	0,678444	1,699031	0,005215	0,020421	0,012104	5074
rno03050	Proteasome	47	-0,68223	-2,45082	0,005495	0,021259	0,0126	6532
rno04211	Longevity regulating pathway	89	0,508667	1,519338	0,005574	0,021313	0,012632	6755
rno04940	Type I diabetes mellitus	61	0,530153	1,521745	0,005787	0,02187	0,012962	6446
rno04024	cAMP signaling pathway	212	0,422712	1,346463	0,006141	0,022941	0,013598	6674

rno04152	AMPK signaling pathway	123	0,477125	1,471605	0,006316	0,023325	0,013825	7381
rno05412	Arrhythmogenic right ventricular cardiomyopathy	77	0,510126	1,501797	0,006865	0,025069	0,014859	5837
rno04218	Cellular senescence	178	0,446984	1,424837	0,007277	0,026276	0,015574	7299
rno05226	Gastric cancer	148	0,454277	1,432438	0,007361	0,026288	0,015581	8235
rno05220	Chronic myeloid leukemia	78	0,509248	1,518601	0,007928	0,028005	0,016599	9005
rno05170	Human immunodeficiency virus 1 infection	227	0,41192	1,339554	0,008163	0,028528	0,016909	6755
rno04662	B cell receptor signaling pathway	77	0,508656	1,524425	0,008909	0,030185	0,017891	6651
rno00970	Aminoacyl-tRNA biosynthesis	67	-0,61897	-2,31985	0,008929	0,030185	0,017891	706
rno01230	Biosynthesis of amino acids	78	-0,45163	-1,73591	0,008929	0,030185	0,017891	7911
rno04136	Autophagy - other	32	-0,51315	-1,7082	0,009009	0,030185	0,017891	8452
rno04913	Ovarian steroidogenesis	56	0,536886	1,529986	0,009456	0,03136	0,018588	5763
rno04915	Estrogen signaling pathway	133	0,453786	1,42921	0,009554	0,031365	0,01859	7241
rno04925	Aldosterone synthesis and secretion	95	0,484997	1,469954	0,009783	0,031793	0,018844	7048
rno00310	Lysine degradation	63	0,504625	1,467656	0,010753	0,0346	0,020508	6633
rno05203	Viral carcinogenesis	211	0,420045	1,359196	0,011168	0,035493	0,021037	8620
rno04918	Thyroid hormone synthesis	73	0,509293	1,484946	0,011249	0,035493	0,021037	5769
rno04068	FoxO signaling pathway	132	0,445318	1,387697	0,011591	0,036222	0,021469	6651
rno05225	Hepatocellular carcinoma	178	0,417989	1,32382	0,013499	0,041784	0,024766	8235
rno04014	Ras signaling pathway	229	0,405584	1,299146	0,014315	0,04389	0,026014	7905
rno01521	EGFR tyrosine kinase inhibitor resistance	80	0,497348	1,489694	0,014509	0,044069	0,02612	7414
rno04371	Apelin signaling pathway	138	0,442529	1,396966	0,014675	0,044161	0,026175	7113
rno01200	Carbon metabolism	122	-0,48092	-1,99231	0,016393	0,04888	0,028972	7911
rno05160	Hepatitis C	156	0,419944	1,338312	0,016895	0,049918	0,029587	9027
